# ParentChecker: a computer program for automated inference of missing parental genotype calls and linkage phase correction

**DOI:** 10.1186/1471-2156-13-9

**Published:** 2012-02-23

**Authors:** Zhiqiu Hu, Jeffrey D Ehlers, Philip A Roberts, Timothy J Close, Mitchell R Lucas, Steve Wanamaker, Shizhong Xu

**Affiliations:** 1 Department of Botany & Plant Sciences, University of California, Riverside, CA 92521, USA; 2 Department of Nematology, University of California, Riverside, CA 92521, USA

**Keywords:** genetic mapping, haplotype, genetic markers

## Abstract

**Background:**

Accurate genetic maps are the cornerstones of genetic discovery, but their construction can be hampered by missing parental genotype information. Inference of parental haplotypes and correction of phase errors can be done manually on a one by one basis with the aide of current software tools, but this is tedious and time consuming for the high marker density datasets currently being generated for many crop species. Tools that help automate the process of inferring parental genotypes can greatly speed the process of map building. We developed a software tool that infers and outputs missing parental genotype information based on observed patterns of segregation in mapping populations. When phases are correctly inferred, they can be fed back to the mapping software to quickly improve marker order and placement on genetic maps.

**Results:**

ParentChecker is a user-friendly tool that uses the segregation patterns of progeny to infer missing genotype information of parental lines that have been used to construct a mapping population. It can also be used to automate correction of linkage phase errors in genotypic data that are in ABH format.

**Conclusion:**

ParentChecker efficiently improves genetic mapping datasets for cases where parental information is incomplete by automating the process of inferring missing genotypes of inbred mapping populations and can also be used to correct linkage phase errors in ABH formatted datasets.

## Background

Lack of knowledge of the parental phase of all alleles segregating in mapping populations can impinge on the accuracy of genetic maps. Recombinant inbred line (RIL) populations developed from two inbred lines are a powerful resource for construction of genetic linkage maps. However, it is not uncommon to observe segregation of markers in RILs that are observed to be fixed in the putative inbred parents of the RIL, and conversely, to observe markers that are polymorphic in the two RIL parents, but fixed in the RIL population. This indicates that the real parents used in the cross to develop the RIL population are different than the available "off parents". This situation probably has two primary causes: 1) where one or both parents were not completely inbred at the time the population was initiated, or 2) from the existence of residual genetic variation within one or both parental lines. This observation is not surprising given that ten or more years can pass between the time when a RIL population is initiated with a cross between two parent plants and the time when it is genotyped along with the presumed parental lines. In both scenarios, one plant of an inbred line was used for the initial hybrid, while another closely related plant of the same inbred line was used for genotyping years later. Thus for case 1) where the original parent plant was heterozygous (Aa) at some fraction of its genome at the time of crossing and then subsequently maintained by inbreeding, the current (more inbred) version of the 'parent' line will have become fixed randomly for either AA or aa, causing the 'unexpected' segregation in the RIL half of the time. For case 2, it is not hard to envisage the existence of limited genotypic differences among individuals within an inbred crop line or variety because it has been standard practice to produce foundation seed-stocks of new cultivars from 'headrow' bulks of 'on-type' highly inbred sublines [1]. Residual genetic variation in homozygous form will be captured in the bulk constituting the Breeder's Seed of such cultivars that can then manifest itself in genetic differences between an individual selected as a parent for RIL population development and another individual of the same line or cultivar that is genotyped.

In other cases, the original parental seed source used to make the RIL population may have been lost as a result of error or project discontinuity, such as personnel changes, which may further complicate the identity of the real parent(s). The problem for the production of a genetic map is that it is advantageous to know the parental phase of all alleles, but the "off-parent" genotypes cannot be used to infer the allele phase of every marker. This "off-parent" problem is most severe when the alleles of both parent stocks are opposite from the alleles in the actual parents of the initial F_1 _plant. However, as long as the genotyped parental stocks are genetically very similar to the actual parents, enough information resides in the mapping population to correctly infer the haplotype composition of the actual parents.

Prior to the advent of high density genotyping, lack of marker coverage limited the prospects to detect cases of the off-parent problem and to correctly infer the actual parent. High-density genotyping greatly increases the opportunity to observe the off-parent problem and enables the inference of actual parental genotypes [2,3]. The increased number of inferences needed with high density genotyping data sets speaks to the need for tools that automate the process of parental inference.

Here we present a new software package, ParentChecker, that addresses two common needs in the preparation of genotyping data for mapping with inbred populations in plant species: 1) inference of the actual parental haplotype, which is relevant to biallelic or ACGT format datasets, and 2) automatic correction of the phase of markers in individuals in the mapping population if the markers are expressed in biallelic format and the parental genotypes are unknown.

## Implementation

The current version of ParentChecker was developed to handle single-nucleotide polymorphism (SNP) data (in ACGT format). However, it also works for other co-dominant markers that are coded in A, B, H or AA, AB, BB format. ParentChecker is very efficient in terms of memory storage and computational speed. On a desktop computer with CPU 2.0 GHz/2 GB RAM, ParentChecker only needs a few seconds to process genetic data from a relatively large segregating population (e.g., 500 individuals with 1000 SNPs). The algorithms implemented by ParentChecker to infer the unknown parental genotypes and linkage phase are as follows:

### Parental genotype inference

Parents used to derive inbred mapping populations are usually assumed to be pure lines. In practice, the parents are often heterozygous for some limited number of loci. Table 1 shows three types of gene transmission patterns for a polymorphic marker when a RIL population is derived. Initially, most loci are heterozygous for both parents. The segregation ratio for genotypes AA:Aa:aa is 1/4:1/2:1/4 for an F_2 _population. The ratio becomes 3/8: 2/8: 3/8 for an F_3_. For each additional generation of selfing, the proportion of heterozygotes is reduced by half and the reduced part is equally divided and added to the two homozygotes. Therefore, the theoretical ratio between the two homozygotes is always 1:1. However, there is no theoretical genotype proportion for the two homozygotes when the cross is made by crossing a homozygote and a heterozygote during the construction of the population. Therefore, a *χ*^2 ^test can be used to determine whether the expected proportions of homozygous individuals are statistically different than 1:1 and thus infer the cross type (e.g. whether it was AA × aa or Aa × aa) for the parents by comparing the observed genotype proportions and the theoretical values listed in Table 1 When a small population is obtained in an advanced generation, the decreasing proportion of the heterozygotes will cause bias to the statistics. Therefore, a special algorithm is needed to adjust for this bias. In ParentChecker, two statistical tests were used to infer the parental genotype: (a) calculating the statistical test for the ratio of two homozygotes against the theoretical ratio of 1:1, which can be calculated by $\chi^{2}={\left(P_{AA}-P_{aa}\right)}^{2}/\left(P_{AA}-P_{aa}\right)\sim \chi_{\gamma =1}^{2}$; (b) calculating the statistics for the ratio the major homozygote to the sum of the other two genotypes against the theoretical ratio defined as

**Table 1:** Theoretical proportions of genotypes in segregating populations generated over 1, 2, 3 and n generations of selfing

	*AA × aa*			*Aa *× *Aa*			*AA *× *Aa*		
**Population**	***p*_AA_**	***p*_Aa_**	***p*_aa_**	***p*_AA_**	***p*_Aa_**	***p*_aa_**	***p*_AA_**	***p*_Aa_**	***p*_aa_**

F_1_	0	1	0	1/4	1/2	1/4	1/2	1/2	0

F_2_	1/4	1/2	1/4	3/8	1/4	3/8	5/8	1/4	1/8

F_3_	3/8	1/4	3/8	7/16	1/8	7/16	11/16	1/8	3/16

F_x_	$\frac{1}{2}-\frac{1}{2^{x}}$	$\frac{1}{2^{x-1}}$	$\frac{1}{2}-\frac{1}{2^{x}}$	$\frac{1}{2}-\frac{1}{2^{x+1}}$	$\frac{1}{2^{x}}$	$\frac{1}{2}-\frac{1}{2^{x+1}}$	$\frac{3}{4}-\frac{1}{2^{x+1}}$	$\frac{1}{2^{x}}$	$\frac{1}{4}-\frac{1}{2^{x+1}}$

F*_n _*(*n *→∞)	1/2	0	1/2	1/2	0	1/2	3/4	0	1/4

(1) $$P_{\text{homozygous1}}:P_{\text{homozygous2 + heterozygotes}}=\left(\frac{3}{4}-\frac{1}{2^{x+1}}\right):\left(\frac{1}{4}+\frac{1}{2^{x+1}}\right)$$

where the major homozygote is defined as the homozygote with frequency higher than that of the other homozygote. The test statistics is calculated as

(2) $$\chi^{2}=\frac{{\left(O_{\text{homozygous1}}-\left(\frac{3}{4}-\frac{1}{2^{x+1}}\right)\times N\right)}^{2}}{\left(\frac{3}{4}-\frac{1}{2^{x+1}}\right)\times N}+\frac{{\left(O_{\text{homozygous2 + heterozygotes}}-\left(\frac{1}{4}+\frac{1}{2^{x+1}}\right)\times N\right)}^{2}}{\left(\frac{1}{4}+\frac{1}{2^{x+1}}\right)\times N}\sim \chi_{\gamma =1}^{2}$$

where *N *is the population size, *O*_homozgous1 _and *O*_homozgous2+ heterozygotes _are observed frequencies for major homozygote and that of the other two genotypes, respectively. ParentChecker assigns the cross type with the smallest statistics value. If (a) is accepted, the segregating population is assumed to be derived from homozygous parental genotypes; otherwise, the initial cross is assumed to have been made between a homozygote and a heterozygote. Although a cross between two heterozygotes can also produce the same ratio as (a), ParentChecker only suggests the cross type of two homozygotes because the probability of a mating between two heterozygotes can be assumed to be very low for known inbred lines of self-pollinated species and safely ignored. In addition, the initial step for cross type Aa × Aa can also be regarded as the cross between two F_1 _individuals. Therefore, there is no fundamental difference between Aa × Aa and AA × aa, especially for advanced generations.

### Linkage phase inference

Consider three adjacent markers that are dispersed along a linkage group as follows:

During meiosis, the frequency of crossovers for each interval is assumed to be independent of other intervals, which means that the recombination frequency between two adjacent markers depends only on the interval size bracketed by the two markers and is not affected by other intervals. Therefore, only the genotypic information of the two markers is relevant for the inference of the linkage phase. This feature allows the use of a hidden Markov model.

Assume that the two alleles for marker M1 are A and a and the two alleles for marker M2 are B and b. The parental haplotype for generating the segregating population is either in coupling phase (AABB and aabb) or in repulsion phase (AAbb and aaBB). Since the linkage phase is a dichotomous event, we consider the coupling phase as status 1 and the repulsion phase as 0. If the hypothesis of coupling phase is rejected, the repulsion phase is accepted.

Frequencies of the two-locus genotypes are listed in Table 2. Gametes that generate the individuals of the mapping population are grouped into four categories: (I) parental type Χ parental type; (II) parental type Χ recombinant type; (III) recombinant type Χ parental type; and (IV) recombinant type Χ recombinant type. Since the frequencies of types II and III are not affected by the linkage phase and the double heterozygote frequencies are identical in types I and IV, the four genotypes (AABB, aabb, AAbb, and aaBB) as shown in the diagonal of Table 2.

**Table 2:** Genotypes formed by gametes and their frequencies under the coupling phase hypothesis.

		Gamete 2			
	
Gamete 1		Parental type		Recombinant type	
	
		AB	ab	Ab	aB
	
		(1-*r*_1_)/2	(1-*r*_1_)/2	*r*_1_/2	*r*_1_/2
Parental type	AB	AABB	AaBb	AABb	AaBB
	
	(1-*r*_1_)/2	(1-*r*_1_)^2^/4	(1-*r*_1_)^2^/4	(1-*r*_1_)*r*_1_/4	(1-*r*_1_)*r*_1_/4
	
	ab	AaBb	aabb	Aabb	aaBb
	
	(1-*r*_1_)/2	(1-*r*_1_)^2^/4	(1-*r*_1_)^2^/4	(1-*r*_1_)*r*_1_/4	(1-*r*_1_)*r*_1_/4

Recombinant type	Ab	AABb	Aabb	AAbb	AaBb
	
	*r*_1_/2	(1-*r*_1_)*r*_1_/4	(1-*r*_1_)*r*_1_/4	*r*_1_^2^/4	*r*_1_^2^/4
	
	aB	AaBB	aaBb	AaBb	aaBB
	
	*r*_1_/2	(1-*r*_1_)*r*_1_/4	(1-*r*_1_)*r*_1_/4	*r*_1_^2^/4	*r*_1_^2^/4

Table 2 is used to infer the linkage phase of the two markers. Although the linkage phase can be investigated by comparing the observed ratio of the parental genotypes to the recombinant genotypes with the theoretical ratio calculated from the length of the interval, a more convenient approach is to test directly whether the observed frequency of the parental genotypes is larger than that of the recombinant genotypes. The null hypothesis is *P*_p _= *P*_r _= 0.5 while the alternative hypothesis is *P*_p _>*P*_r_,

where

(3) $$P_{p}=\frac{P\left(AABB+aabb\right)}{P\left(AABB+aabb\right)+P\left(AAbb+aaBB\right)}=\frac{{\left(1-r_{1}\right)}^{2}}{1-2\left(1-r_{1}\right)r_{1}}$$

and

(4) $$P_{r}=\frac{P\left(AABB+aabb\right)}{P\left(AABB+aabb\right)+P\left(AAbb+aaBB\right)}=\frac{r_{1}^{2}}{1-2\left(1-r_{1}\right)r_{1}}$$

The recombination frequency between M1 and M2 is denoted by *r*_1 _and is calculated from dl using Haldane's [4] or Kosambi's [5] map function. The null hypothesis can be tested using $\chi^{2}={\left(P_{p}-P_{r}\right)}^{2}/\left(P_{p}+P_{r}\right)\sim \chi_{\nu =1}^{2}$. However, in practice, calculating the test statistics is unnecessary for linkage phase inference even if the interval size is relatively large. For example, let the distance between M1 and M2 be 30 cM, the theoretical values for *P*_p _and *P*_r _and are 0.9218 and 0.0782, respectively. Suppose that there are only 50 individuals in total for the four genotypes in the diagonal of Table 2 in the segregating population. Even if the observed numbers of individuals for AABB + aabb and AAbb + aaBB are 35 and 15, respectively, the statistical test is still significant because the *p*-value is 0.0006. Therefore, if the observed counts for AABB + aabb are larger than that of AAbb + aaBB, it is statistically safe to suggest that the linkage between M1 and M2 is coupling if the observed *P*_p _is larger than *P*_r _without calculating the test statistics.

## Results and discussion

ParentChecker uses the segregating patterns of markers and a linkage map to infer the parental genotypes that produced the segregating population. The formulas that are implemented in the current release of ParentChecker rest on two assumptions: the molecular markers are codominant and markers exhibiting distorted segregation have been removed from the dataset. Users are strongly suggested to use the built-in functions of ParentChecker to remove incompetent markers from the genotypic data before exporting the final outputs. Although the fundamentals of phase inference in linkage analysis has been discussed in detail [6-9], the strategy employed in ParentChecker in handling phase issues is slightly different from other approaches. We used the Chi-square test in an intuitive way instead of a maximum likelihood method and implemented this by an expectation-maximum algorithm, to infer the linkage phase. It only requires a minimal amount of calculation, which is helpful for handling high density SNP data. Furthermore, it offers a convenient way to determine the correct linkage phase at a high level of statistical confidence without requiring actual calculation of test statistics.

For SNP data, a recommended workflow for ParentChecker would be to load data in ACGT format and use the output information (inferred parent) from ParentChecker for subsequent analysis such as building improved maps and QTL detection. For SNP data inputted in ACGT format, ParentChecker can generate an output in ABH format suitable for mapping and QTL detection. Furthermore, ParentChecker can directly export input files for popular genetic software packages including FlapJack [10], GGT [11], MapQTL [12], PowerMarker [13], Structure [14], and Tassel [15]. For other types of molecular marker data (e.g. SSRs) that are coded in ABH format, ParentChecker can be used to automatically correct linkage phase errors, which may be caused by missing values and genotyping errors [16] in parental genotypic data. But unlike Joinmap [17], FlapJack [10] and GGT [11], ParentChecker automatically recodes the genotypic data according to the linkage phases it inferred and a user interference is not necessary.

The input data format for ParentChecker is flexible. ParentChecker can take data directly from tab-delimited text files or import data from an Excel clipboard. The order of the markers in the genotype file does not have to match the order of the markers in the map as long as the marker names are consistent between the two files.

ParentChecker efficiently improves mapping datasets for cases where parental information is incomplete. The observation of missing parental haplotypes in the development of a consensus map of cowpea [18] spurred the development of ParentChecker. The consensus map was constructed by merging individual maps made from 11 RIL and 2 F_4 _mapping populations that had been genotyped with the Illumina 1536-SNP GoldenGate Assay [19]. Nine of the 11 RILs and both F_4 _populations had at least one case of missing parental genotype information, with the number of missing parent data totalling 310 instances and ranging from 1 to 107 per mapping population (Table 3). An iterative process was employed which included detecting suspicious linkage phases using JoinMap4, correcting the linkage phase errors manually, and re-checking the parental phase visually with FlapJack. This tedious one-by-one process produces correct phase designations, however, it requires user-based decisions which are time consuming and which can be subjective. Of the 310 additional SNP data points where phase was assigned arbitrarily, one-hundred and forty-eight, or approximately half, required phase reversal. Using the manual method with JoinMap4 potential linkage maps had to be generated each time a marker exhibited characteristics of an uncertain parental phase. These potential maps were then checked within JoinMap4 [17] and visually through FlapJack [10] and re-mapped, if necessary. The process required numerous iterations until a satisfactory fit was obtained and parental phase finally assigned. ParentChecker is able to accomplish this task in less than 2 minutes. Given the large datasets currently being generated in many crops by high-throughput genotyping platforms, there is a need for the automation of parental inference and data export flexibility provided by ParentChecker.

**Table 3:** An excerpt from Lucas et al. 2011 [18]

Population	Individuals	Mapped SNPs	SNPs Phase Unknown	SNPs Phase Reversed
524B × IT84S-2049	85	438	0	0

CB27 × 24-125B-1	87	329	0	0

CB27 × IT82E-18	160	430	27	10

CB27 × IT97K-566-6	92	438	16	7

CB27 × UCR 779	56	560	51	26

CB46 × IT93K-503-1	114	374	17	10

Dan Ila × TVu-7778	79	288	107	46

IT84S-2246 × IT93K-503	88	155	22	11

IT84S-2246 × Mouride	87	347	60	32

LB30#1 × LB1162 #7	90	180	1	0

Sanzi × Vita 7	122	413	5	3

TVu14676 × IT84S-2246-4	136	345	4	3

Yacine × 58-77	97	435	0	0

## Conclusions

ParentChecker is an automated tool designed to efficiently infer parental genotypes for improved map resolution. It also helps researchers to recode genotypic data to match the underlying linkage phase of RIL populations.

## Availability and requirements

Project name: ParentChecker

Project home page: http://statgen.ucr.edu/software.html

Operating system(s): Windows XP/7

Programming language: Delphi

License: Freeware

Any restrictions to use by non-academics: None

Additional materials: Two sample datasets from our cowpea project are provided in the ParentChecker package for testing and demonstration purposes.

## Competing interests

The authors declare that they have no competing interests.

## Authors' contributions

ZH designed and implemented the software used in this project. JDE conceived of the study, participated in the design of the application and helped draft the manuscript. PAR, TJC, SW, ML and SX participated in the design of the application and helped draft the manuscript. All authors read and approved the final manuscript.

## Authors' information

ZH is working under the direction of SX conducting research in the analysis of quantitative genetic variation. JDE is a cowpea breeder developing modern breeding methods. PAR, TJC, SW, and ML are conducting genetic research and genomic resource development on cowpea in support of breeding and trait discovery.
